# An ICT Prototyping Framework for the “Port of the Future”

**DOI:** 10.3390/s22010246

**Published:** 2021-12-30

**Authors:** Davide Barasti, Martina Troscia, Domenico Lattuca, Alexandr Tardo, Igor Barsanti, Paolo Pagano

**Affiliations:** 1 Consorzio Nazionale Interuniversitario per le Telecomunicazioni, National Laboratory of Photonic Networks & Technologies (PNTLab), Via Giuseppe Moruzzi, 1, 56124 Pisa, Italy; martina.troscia@cnit.it (M.T.); domenico.lattuca@cnit.it (D.L.); alexandr.tardo@cnit.it (A.T.); igor.barsanti@cnit.it (I.B.); paolo.pagano@cnit.it (P.P.); 2 Department of Information Engineering, University of Pisa, Via G. Caruso, 16, 56122 Pisa, Italy

**Keywords:** port community system, terminal operating system, navigation safety, logistics, e-freight, IoT-based monitoring, connected vessel, 5G mobile communication, smart port

## Abstract

Seaports are genuine, intermodal hubs connecting seaways to inland transport links, such as roads and railways. Seaports are located at the focal point of institutional, industrial, and control activities in a jungle of interconnected information systems. System integration is setting considerable challenges when a group of independent providers are asked to implement complementary software functionalities. For this reason, seaports are the ideal playground where software is highly composite and tailored to a large variety of final users (from the so-called port communities). Although the target would be that of shaping the Port Authorities to be providers of (digital) innovation services, the state-of-the-art is still that of considering them as final users, or proxies of them. For this reason, we show how a canonical cloud, virtualizing a distributed architecture, can be structured to host different, possibly overlapped, tenants, slicing the information system at the infrastructure, platform, and software layers. Resources at the infrastructure and platform layers are shared so that a variety of independent applications can make use of the local calculus and access the data stored in a Data Lake. Such a cloud is adopted by the Port of Livorno as a rapid prototyping framework for the development and deployment of ICT innovation services. In order to demonstrate the versatility of this framework, three case studies relating to as many prototype ICT services (Navigation Safety, e-Freight, and Logistics) released within three industrial tenants are here presented and discussed.

## 1. Introduction

The vast majority of seaports are currently offering digital services to their user communities (the collective name for ocean carriers, hauliers, inter-modal carriers, shippers, freight forwarders, insurance companies, as well as institutional and control bodies). Each port is supposed to set up an information system, known as Port Community System (PCS [1]) connected to the National Single Window, established at the government level for customs and maritime business normative procedures [2]. PCSs are used by private and public institutions to enable intelligent and secure exchange of information, to improve the efficiency and competitive role of seaports, to automate and smooth out port logistics processes through a single submission of data, and by connecting transport and logistics chains. Therefore, PCSs are a commodity appliance whose scope and technical specifications depend on the port nature and is supposed to evolve hand in hand with new requirements coming from the physical and digital development of naval and terrestrial shipping. In the absence of a comprehensive standardization of seaport digital services [3], the best seaports have implemented fully customized solutions to provide their user communities with digital services, often delivered through a (private) cloud. Frontier technologies such as 5G, Distributed Ledger Technologies, IoT, and Artificial Intelligence permit to include these digital services into an extended scope of their PCSs, supporting long-term strategic planning as well as observation and real-time response to critical events, including disasters. The Port Authorities are therefore increasingly shaped as (digital) service providers. As providers, they are then requested to allocate resources (computing power, peripheral equipment such as networks and sensors), to authenticate users, and authorize access to specific data sets. In such a perspective, Port Authorities would benefit from fulfilling the role of cloud service providers, rather than the one of resource proxy. The goal is to rely on a digital framework capable of being scaled, replicated, and adapted to specific configurations. The target framework is therefore a private cloud, structured in tenants, accessible to the user communities; the tenants are standardized as much as possible in terms of the data model, interfaces, and communication protocols. Even more, if the tenant is configured to permit continuous development and agile prototyping, such architectural choice can be defended and encouraged as a future-proof investment. In this paper, we present the development of an ICT framework in Livorno targeting the aforementioned functionalities; the authors consider the actual setup as a promising example in the EU panorama and present the results contained in this paper as a convincing proof of concept. The remainder of this paper is organized as follows. In Section 2, we present the most relevant experiences in the port sector; we will in turn present in Section 3 the Port Monitoring System experimentally implemented in Livorno (embedding the local PCS). We will describe each layer of the Livorno cloud resources and the multi-tenant infrastructure that we have designed. Toward the end of the section, we will go through some applications prototyped to validate the tenant infrastructure. We will finally present some preliminary results in Section 4 and discuss them in Section 5 prior to our conclusions.

## 2. Related Work

In the last few years, integration between inland terminals and hinterland logistics services shifted from the classical regional port paradigm to a more global and interconnected one [4]. Wilmsmeier and Notteboom [5] identified four phases of ports’ role evolution in the shipping service, ranging from predominantly regional and not interconnected, to an overseas shipping service. In this last stage, the larger ports represent a central hub and also considerable support for other nearby smaller ports [6,7]. In addition, the major seaports are improving their PCSs by taking advantage of innovative ICT services to face safety, security, operational, and energy efficiency challenges [8]. The presence of a solid ICT infrastructure at the base of a PCS is the first step in the transition from a classic pre-1990s port [9,10], where the absence of ICT services does not allow the automation of some of the most common port processes (e.g., security-related or logistic), to a new category of seaport, commonly referred as smart port. In this new type of seaports, several operations are automated and monitored according to the principles of self-configuration, protection, healing, and optimization, typical of any informative system that can be defined as “smart”. The next phase envisages the adoption of a global standard approach for ICT stack structuring, which is the foundation for every PCS. Such architecture should be opened in order to facilitate microservices development through all the related layers: data, business, and application. This final step is fundamental to achieve a common ICT reference model for PCSs, and complete the transition from the smart port to the so-called “Port of the Future” [3]. The major worldwide and national organizations, such as the International Maritime Organization (IMO), the United Nations Conference on Trade and Development (UNCTAD), and the European Union (EU), have made great efforts to provide standards to assess port smartness [11]. On the other hand, current literature includes different examples of frameworks relating to the seaport area. Some of them are described below, highlighting pros and cons, and divided accordingly to some critical aspects of ports they deal with. Specifically, the framework categories taken into account, possibly combined, are investments, sustainability, and situational awareness. Each of these frameworks exploits different approaches in order to guide the port governance strategy, for instance, the work in [12] proposes a system to optimize port investments based on a three-stage process: evaluation of the ship’s response to external impacts (e.g., weather conditions variation) in order to establish operational safety policies, model building and simulation to see the impact of different investments on port performance, and generation of the optimal investment planning. In [13], Ignaccolo et al. recommend a framework for actions and measures to foster sustainability in ports using three fundamental sustainability pillars and the Avoid-Shift-Improve (A-S-I) approach [14]. The sustainability paradigm is here based on the following strictly related aspects: environmental, economic, and social. These aspects must be taken into account altogether in order to select the best port strategies, together with improving the decision process. Moreover, Acciaro et al. [15] proposed a framework to efficiently select the initiatives capable to improve the environmental sustainability of the seaports, by prior defining a list of objectives to be validated. The results show an inevitable connection between successful initiatives and policy action taken by the proper authorities. In a later work [16], Stein and Acciaro analyzed a crucial aspect that should drive the port’s strategic decisions, called Corporate Sustainability (CS). Specifically, their paper shows which factors can affect the CS, and how it can improve the port’s competitiveness. Other works, such as those in [17,18], proposed a framework for noise monitoring in response to the rapid increase of operations within the port area. The results highlighted a deficiency of specific regional laws designed to mitigate complaints from people who live near ports. Finally, these works showed an actual difficulty in fulfilling their objectives due to a lack of data on noise impact from the ports and the absence of a shared approach to port noise monitoring. Several works apply the Internet of Things (IoT) paradigm, using connected sensors to monitor environmental parameters from apparently unrelated port processes, with the aim of connecting them to descry new cause–effect relationships. In this regard, the work in [19] suggests a method to obtain situational awareness information about critical port infrastructures. The proposed approach combines information from the cyber and physical domains (e.g., camera and sensors) to prevent cascading effects of incidents. The results proved the presence of a bottleneck in the system capacity to manage heterogeneous data sources from different port assets. In addition, Lacalle et al. [20] devise an IoT-based framework with a methodology for defining, calculating, and predicting composite environmental indicators (e.g., weather or traffic) that represent real-world phenomena in smart port cities. The tests based on real data coming from the port of Thessaloniki (Greece) showed a high level of management and usability of information arising from heterogeneous sources, paving the way for a standardized methodology for port-related data handling. Table 1 summarizes the evaluated frameworks and classifies them according to the seaport aspects addressed.

The above-cited approaches only address a limited set of port issues categories, often without describing the main features of the adopted ICT stack. The informative stack at the base of a PCS should be open, in order to support and facilitate services development. Furthermore, none of the discussed methodologies is standardized or shared at a regional or global level. In a previous work [3], we proposed an ICT service structure for the PCSs able to manage different categories of typical port services (e.g., Vessel and Marine Navigation, e-Freight and Intermodal Logistics, Passenger Transport, and Environmental sustainability) and ready to be used and standardized. In this paper, three examples of real port services are presented to demonstrate the capability of the proposed ICT architecture to be effectively implemented in any size-category port, becoming a best practice for the seaport’s sector.

## 3. The Reference Architecture

**Figure 1:**
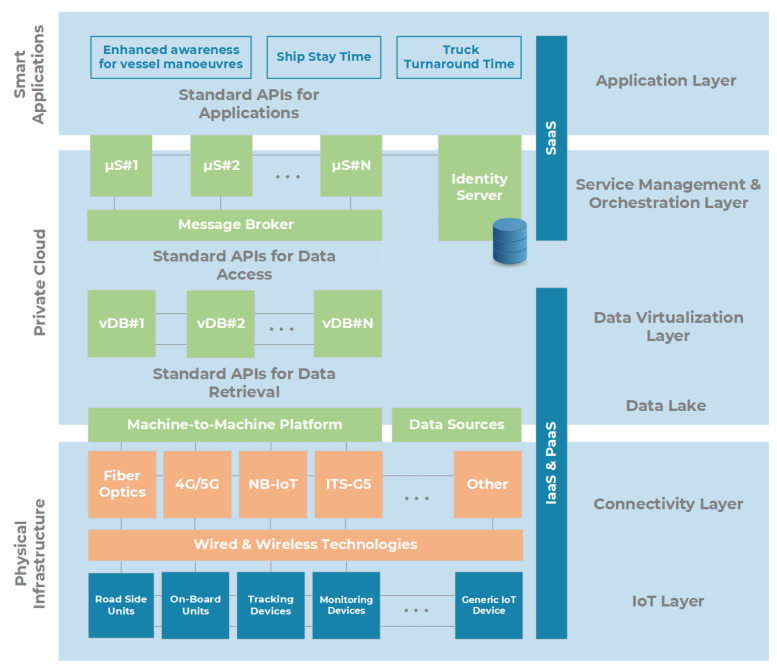
Livorno Port ICT Reference Architecture (MONICA).

In recent years, the Livorno Port Authority has invested in the standardization of an ICT reference stack, named MONICA Standard Platform. The MONICA architecture, represented in Figure 1, is structured as a private cloud with full decoupling of the three canonical layers [21], and it is appointed by the Authority in current and future tenders and procurements. All innovation processes in the work plan of the collaborative projects are integrated into the (experimental) offer of digital services targeted to citizens and industrial communities. The described infrastructure allows the development of PaaS and SaaS functions in the shape of microservices, developed following an agile methodology (see the end of the section), then exposed through software interfaces (APIs). Networks and computing resources in the cloud are organized in tenants serving (group of) users.

**Figure 2:**
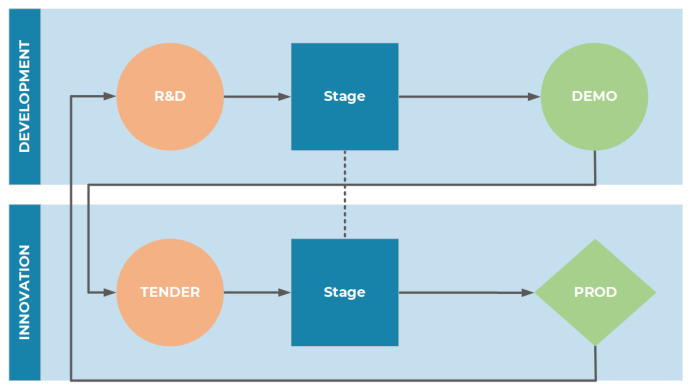
Development Line Concept.

The cloud has been replicated in a local laboratory to permit Test and Validation of the most recent functionalities, prior to the release time. The local cloud is configured as a staging environment for demo applications. According to the concept shown in Figure 2, the demo applications (Development Lane) are turned into funding opportunities for industries (Innovation Lane) allowing them to fill the gap from the prototype to the final product or service.

### 3.1. Cloud Resources

The three canonical layers have been incrementally deployed at the Port of Livorno, encompassing cutting-edge technologies and state-of-the-art commercial solutions.

#### 3.1.1. Infrastructure as a Service Layer

**Figure 3:**
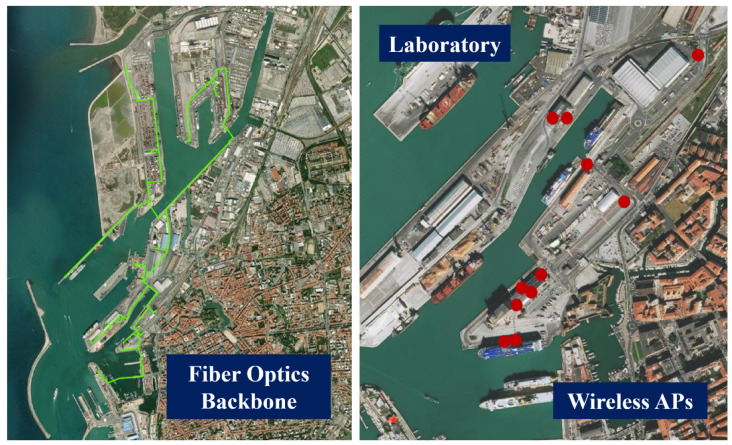
Cabled and wireless network layout of Livorno Port.

The Port of Livorno is fully covered by a fiber optic backbone that capillary serves all terminals and gates in the port area. On one hand, the fiber optic star-shaped network allows connecting all digital resources (i.e., sensors and actuators, network adapters, and ordinary and industrial PCs) to a local LAN. On the other hand, the network mitigates the digital divide suffered by certain areas, permitting to provide Internet Access to government institutions (notably the Coast Guard and the State and Fiscal Police corps), through the ISP selected by the Authority. A complementary wireless backbone is covering the maritime station with 100 Mbps Wi-Fi technology. The network is composed of five nodes and is attached to the lab network through a gateway. The layout of both cabled and wireless networks is shown in Figure 3. In addition, the network serves the headquarters of the active pilot corporation located at the edge of the port sector dedicated to cruise and ferry naval traffic.

In 2016, Livorno was the first port in Italy featuring NB-IoT commercial services. Later on, a prototype 5G network [22] covered part of the container terminal and is now accessible from the laboratory via a dedicated link in the fiber optic backbone. Part of the computer farm in the laboratory is also configured as the edge node running the 5G core network functionalities with the capability of serving prompt communication in real-time applications. This capillary network permits to connect a set of heterogeneous sensing resources to the upper layers of the MONICA information stack presented at the beginning of the section.

Without any sake of completeness, some resources integrated so far include the following:

- a set of Road Side Units (RSUs) and On-Board Units (OBUs) following the EU directives about “Smart Roads” and complying with ETSI TC ITS standards [23];
- a set of smart IoT devices measuring sea current strength and wave height, weather conditions, noise level, presence of water standings on the landside (at the port and in highways/motorways), air contamination (pollutants and GHG, most notably methane and CO${}_{X}$ levels), etc.;
- a Real-Time Kinematics anchor permitting to correct the GNSS signal in a noisy environment (as when in the proximity of container stacks and docked vessels);
- a bathymetric probe (multibeam sonar emitter) to measure the water depth around the vessel trajectories.

#### 3.1.2. Platform as a Service Layer

This layer supervises the data storage, processing, aggregation, and sharing. The main research topic is that of establishing a set of components for Data Lake management capable of

- providing a unique set of APIs for data access, regardless of the specific technology used (either relational, document-based/non-relational, geographical, IoT-oriented, object-oriented, time series, etc.),
- providing mirror databases of external sources of information managed by independent organizations (e.g., road, city, and regional traffic and mobility centers), and
- interoperating with a set of commercial and sector-specific Distributed Ledger Technologies (i.e., blockchains), securing trusted information in a privacy-aware manner.

**Figure 4:**
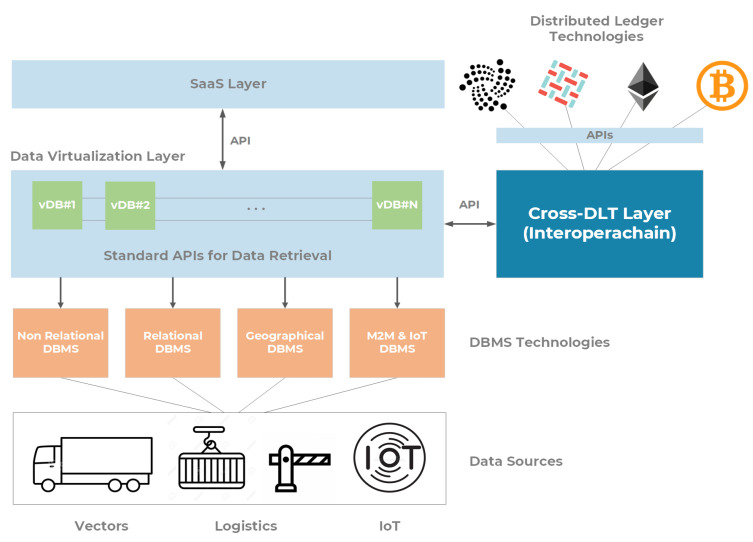
Data Lake implemented at the Port of Livorno.

In Figure 4, a graphical representation of the Platform-as-a-Service layer in the Port of Livorno is shown. In order to provide a proper set of technologies to be used for Data Lake management, we relied on the following solutions.

- JBOSS Teiid—Data Virtualization Layer [24]: a cloud-native and open-source data virtualization platform enabling distributed databases, as well as multiple heterogeneous data sources, to be accessed by means of a common and standard set of APIs (e.g., JDBC, ODBC, REST, OData, SOAP, etc.). On one side, it allows aggregating data coming from disparate data sources, and on the other side, it permits to define a proper set of roles (according to create-read-updated-delete operations) allowing data consumers to consume specific data sets. The current instance of the data virtualization layer is based on the WildFly application server [25], providing robust operations for transaction management, connection pooling, security configuration, resource management, and clustered deployment. The above-mentioned instance is deployed in the form of a docker container, running on a virtual machine using Ubuntu 20.04 Server as the main operating system (64-bit architecture). The following basic configuration of the considered virtual machine has been used: 8 GB of RAM, 100 GB of storage space, and 4 CPU cores (AMD EPYC 7281). In order to feed the developed service prototypes with real data, three different virtual databases and procedures have been implemented at this level.
- Mobius OneM2M IoT Server Platform [26]: an open-source IoT platform based on the oneM2M standard [27]. The considered implementation of the standard supports a resource-oriented architecture with a common set of service functionalities such as registration, discovery, security, groups management, data management, subscription, notification, device management, network service exposure, location, etc. Moreover, the solution supports multiple protocols binding over standard interfaces (e.g., HTTP, CoAP, MQTT, or Web Sockets). The Mobius machine-to-machine platform allows managing and interacting with smart IoT devices deployed in the Port of Livorno, such as meteorological stations, bathymetric probes, pollution and parking sensors, as well as data coming from the Automatic Identification System dispatcher. It has been deployed in a form of a docker container within a dedicated virtual machine with the following configuration: Ubuntu 20.04 Server as the main operating system (64-bit architecture), 8 GB of RAM, 100 GB of storage space, and 4 CPU cores (AMD EPYC 7281).
- Database Management Systems (DBMS): a set of software systems used to define, store, retrieve and manipulate data in a heterogeneous set of databases, serving the current information systems within the Port of Livorno, such as the Tuscan Port Monitoring System (TPCS [28]) and several monitoring applications. This includes relational databases (SQL-compliant), document-oriented databases (e.g., MongoDB), and object-oriented data sources (e.g., PostgreSQL). An adaptation to the ESRI ArcGIS has also been implemented. The considered data sources are then made accessible by means of the Data Virtualization Layer through a common set of wrappers, according to a specific prototyping service.
- Interoperachain—Cross DLT Layer: a mediator service providing data immutability capabilities by interacting with different distributed ledger technologies such as Bitcoin, Ethereum, HyperLedger Fabric, and IOTA (the main distributed ledger technology used at the Port of Livorno). It has been implemented by using the OpenAPI standard for the input/output interfaces definition that can be then easily integrated, abstracting the complexity of the underlying DLTs. As a matter of fact, OpenAPI allows to automatically generate client-side code by supporting 33 different programming languages. Interoperachain guarantees a high service availability as well as a robust fault tolerance, avoiding technological lock-in from the user perspective.

#### 3.1.3. Software as a Service Layer

**Figure 5:**
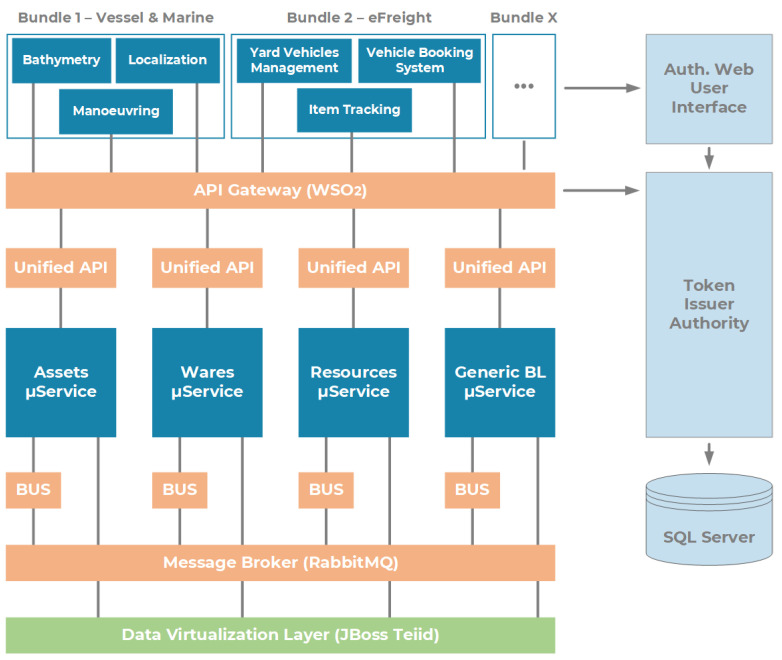
A microservice-based architecture for the SaaS layer of the MONICA architecture.

The layer consists of two stacked blocks of services devoted to software orchestration and front-end applications. A microservice-based approach for software development has been adopted: it allows agile API development, life cycle management, and access according to the specific end user. A centralized and resilient software stack for data lake access has been implemented by the Livorno Port Authority, adapting it for the MONICA architecture. The framework is shown in Figure 5.

This solution allows for seamless software composability, regulated access to orthogonal data sets, a clear distinction between technical background processes, and front-end interfaces intended for final users. In the figure, such microservices have been pictorially labeled as related to port assets, wares, digital resources, and business logic aimed at data aggregation and knowledge extraction. It is possible to aggregate any self-consistent logic into containers and virtual machines, as it will be presented later in the section.

The Software-as-a-Service layer implements typical Enterprise Service Bus (ESB) functionalities, and it consists of the following open-source software-based solutions that we relied on:

- WSO2 API Gateway [29]: an open-source, standardized, and componentized middleware platform that implements typical enterprise service bus functionalities by supporting microservices’ logic development (by means of SDK based on ASP.NET Core Framework) and APIs’ life cycle management. It secures, protects, manages, and scales API calls by intercepting API requests and applying security policies. The WSO2 API Gateway instance is deployed in a docker container, running in a virtual machine and using Ubuntu 20.04 Server as the main operating system (64-bit architecture), 4 GB of RAM, 80 GB of storage space, and 2 CPU cores (AMD EPYC 7281).
- RabbitMQ [30]: an open-source and multiprotocol message broker supporting the communication and interoperability among different microservices. Based on asynchronous communication, it allows microservices to perform distributed tasks by communicating with each other throughout high-performance queues. RabbitMQ instance has been deployed in a dedicated virtual machine with Ubuntu 20.04 Server as operating system, 4 GB of RAM, 40 GB of storage space, and 1 CPU core (AMD EPYC 7281).
- Authorization Server: authentication and authorization component of the ICT stack, based on industry-standard protocol OAuth2 [31]. According to this, we relied on OpenID Connect 1.0 Hybrid Flow [32] as the identity layer on top of the OAuth 2.0 protocol. This allows clients to verify the identity of the end users based on the authentication performed by the Authorization Server, as well as to obtain basic profile information about such end users in an interoperable and REST-like manner. The Authorization Server implements a single-sign-on (SSO) authentication scheme, and it is built upon the authorization token (JSON Web Token-JWT [33]) which is released to a specific user in order to invoke selected microservices. The JWT is emitted and validated by a token issuer authority, according to a given, as well as predefined, set of grants and access rules. In our configuration, the Authorization Server is deployed in a virtual machine whose specifications are based on Ubuntu 20.04 Server as an operating system, 4 GB of RAM, 80 GB of storage space, and 2 CPU cores (AMD EPYC 7281).

**Figure 6:**
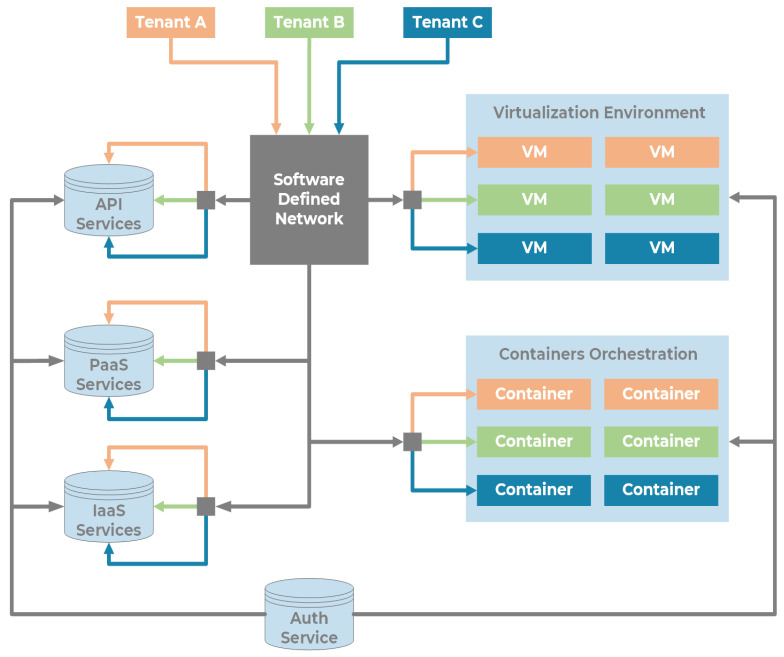
Multi-tenant infrastructure.

We provide a system architecture capable of offering heterogeneous services, usable by multiple tenants that access the system adopting different network technologies directly, or in a private way through a VPN. In order to deploy a tenant profiled to a set of independent users, we offer a Software-Defined Network and a centralized Authentication, Authorization, and Accounting (AAA) service to manage permissions to access or use a specific part of the software stack and data lake. Each tenant accesses services through different paradigms (possibly in combination): API endpoints to access services and data; machine virtualization, enabling tenants to deploy their servers on dedicated network segments; containers orchestration environments, to allow tenants to deploy containers. The multi-tenant infrastructure is shown in Figure 6.

In order to allow agile management and orchestration of the hosting resources, we relied on the following tools:

- Swagger [34] is an OpenAPI framework. Inside the Swagger environment, it is possible to design API basic code using YAML files that describe API behaviour and contain the API documentation. The Swagger framework produces also a code skeleton for the interface.
- VMWare ESXi [35] is a virtualization hypervisor. ESXi manages multiple Virtual Machines connected through Software-Defined Networks.
- Kubernetes [36]: some infrastructure components run in docker containers. Kubernetes manages the containers and creates, deploys, and stops instances based on multiple configurations files;
- Software Defined Networking (SDN): the network is configured with software switches, VLANs, and firewalls, inside the VMWare network layer and the Kubernetes virtual network.

### 3.2. Design and Development of Service Prototypes

In this subsection, we finally describe the design and development of three prototypes to prove the versatility of the stack widely described previously. The MONICA platform unlocks the ability to freely interact with the three layers that define a typical application: data, business, and application. The limits of classical port systems, where closed, proprietary solutions are omnipresent, are overcome with an accessible and standardized stack.

The prototypes have been selected based on the priority imposed by the Port Authority, and presented in the Port Master Plan in force [37]. This official document explicitly envisages developing new digital solution addressing (i) “Rail–road and rail–sea integration (inter-modality) for people and goods”, (ii) “Navigation aid system for assistance to pilots and maritime authorities”, as well as (iii) “Integrated environmental monitoring and control of the port-logistics processes”. The proposed *Service A* is addressing topic (ii), while *Service B* and *Service C* are addressing topics (i) and (iii).

#### 3.2.1. Service A: Enhanced Awareness for Vessel Manoeuvres

This prototype microservice has been built in the context of the European H2020 project PortForward [38]. The project aims to address important challenges that modern ports are facing, such as lack of efficiency in operations with heterogeneous freights, need for real-time monitoring of freight flows and remote management of important port operations, need for an interface with the surrounding urban environment, and environmental impact reduction through the use of green technologies and energy-saving solutions. PortForward addresses these problems through several tools, such as sensor deployment and interconnection into a versatile and secure IoT network, remote management and smart logistic platform with Decision Support System (DSS), Augmented Reality (AR), environmental and energy monitoring/optimization system using Green Scheduling (GS). More specifically, this prototype microservice allows retrieving heterogeneous information about a ship and the surrounding environment to assist pilots during the vessel manoeuvres in the port of Livorno. Pilots are mandatory in all seaports in Italy, and they are in charge of safe and secure maneuvering of all vessels within the harbor. Typically, they are part of on-board personnel assisting captains of the vessels and providing maneuvering instructions. They do not have access to real-time information beyond the visual horizon, but they have to rely on their expertise or get crucial information from port authorities by radio. This prototype provides in a single place all the required information. The microservice needs to collect data from a variety of heterogeneous sources, but this operation has been particularly easy thanks to the microservice oriented stack. The data virtualization layer allows easy and standard retrieval of the information adopting the OData protocol (through RESTful APIs implementation), while data are physically stored in databases implemented with different technologies and IoT-oriented platforms (e.g., Mobius OneM2M platform [27,39]). The user can retrieve real-time data including the position of the vessel, as well as its speed and direction, “static” information concerning the vessel (e.g., IMO, dimensions, type), information about the surrounding infrastructure (e.g., channel width and depth) and other nearby ships, bathymetries, and weather information. The Graphical User Interface (GUI) consists of a pair of Hololens smart glasses able to interact with the prototype microservice. The glasses react to vocal commands and show the information previously described. In this way, the pilot can combine the visual information with data from the stack and make better-informed decisions.

#### 3.2.2. Service B: Ship Stay Time

The Ship Stay Time (SST) prototype provides end users with information regarding the *mooring time* of a ship, meaning the time spent inside the port. The port information system registers the events of the ship entering and leaving the Port of Livorno, and the current infrastructure allows to seamlessly obtain and integrate data in the end user application. With the available data, it is possible to group the events by ship and present to the end users such data in a way that they can easily exploit to examine the overall efficiency and possibly plan for further improvement of the process. Access to this kind of data allows to quickly identify inefficiencies in the current scenario and act accordingly. Being able to spot crucial points in time when something goes wrong is a precious resource for domain experts that can take control of the situation and possibly take actions to mitigate the problem. Reducing the SST can positively affect the environmental impact of the Port (a ship that docks for less time will likely pollute less) but also the costs (reducing the SST means being able to handle more ships). In practice, the user will use the IMO identification number to search for data relative to a specific ship. If the IMO number is valid (in the context of the application, the IMO number is valid if the information system contains data relative to the specified ship), the application will return information about both the latest available ship stay time and historical data visualized in a graph. The user also has the possibility to restrict the search to a specified period of time. Following the microservice architecture described at the beginning of the section, the application references the database through a dedicated DVL node. The virtualization of the underlying data allows independent access to storage, without any concern on the actual database type and the relative data format. The core part of the prototype is the microservice that provides a clean and well-defined interface to the end user web application (API), that accesses the Teiid virtual database using the OData protocol, serving data to the upper layers of the architecture. The microservice provides two main features: retrieval of the latest data available for a ship and retrieval of the historical data, respectively, the *GetShipStayTimeLatest* and *GetShipStayTime* endpoints. The Graphical User Interface has been designed to allow end-users to access the data, simply providing the IMO number of the ship they are interested in. The system will seamlessly access and collect the data, and show it to the user on the screen. The clean and well-documented design of the SST microservice allows access to the underlying data in a simple and straightforward way.

#### 3.2.3. Service C: Truck Turnaround Time

The Truck Turnaround Time prototype provides end users with information regarding the time spent by a given truck within the seaport area. Typically, when a truck arrives at the port entrance, the plate and container number (if present) is read and if the vehicle is authorized to enter the port facilities, the date and time of access are registered (*gate-in event*), and the barrier is lifted. After the loading/unloading operation, trucks leave the terminal and the port facilities: again, the plate and container number (if present) is read and if the vehicle is authorized to leave the port facilities, the date and time of exit are registered (*gate-out event*), and the barrier is lifted. The difference between the moment the truck enters the port facilities and the moment it exits the port facilities is called Truck Turnaround Time (TTT). The knowledge of TTT can pave the way to different methods oriented to optimize the access to the port facilities and/or reduce the waiting time for vehicles at the port gate, leading to corresponding savings on direct costs for carriers. For example, specific applications can make use of TTT to develop a vehicular booking system to minimize waiting times, ensure more efficient operations, and reduce emissions caused by truck congestion. Even more, a web application can help to deliver visual information using machine/deep learning algorithms for future TTT predictions and analysis (e.g., predictive models for the identification of factors that may affect the TTT). To handle all of this information, we adopted the microservice oriented architecture described at the beginning of the section. The microservice retrieves the data from the DVL adopting the OData protocol [40] and elaborates the information, making it available for the application layer. A user previously authorized by the stack authentication system can have access to the turnaround time of a specific truck. The truck is identified by its anterior plate, that is, the plate of its tractor. The Graphical User Interface is populated through the invocation of the methods exposed by the APIs of the aforementioned microservice. In particular, the user can read the most recent TTT related to the truck (obtained by the *GetLatestTTT* endpoint of the microservice). They can also obtain historical TTT data in a graphical form (obtained from the *GetTTTHistory* endpoint of the microservice). Future developments of the GUI can include the possibility to limit the TTT “history” to a specified time interval, exploiting the *GetTTTHistoryInInterval* endpoint of the microservice.

## 4. Results

**Figure 7:**
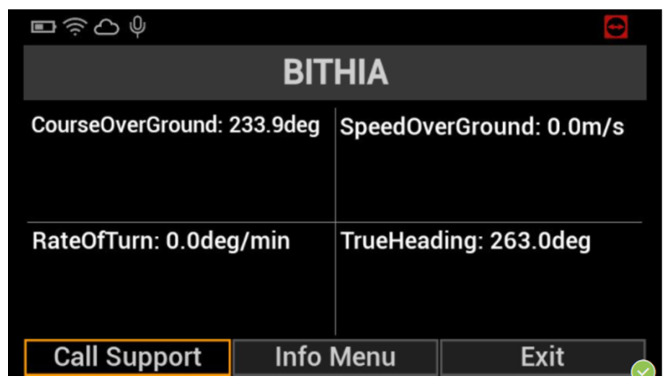
Real-time information shown by the smart glasses.

**Figure 8:**
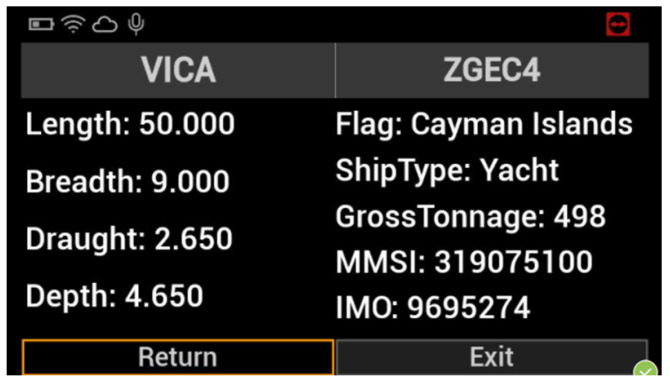
Static information shown by the smart glasses.

**Figure 9:**
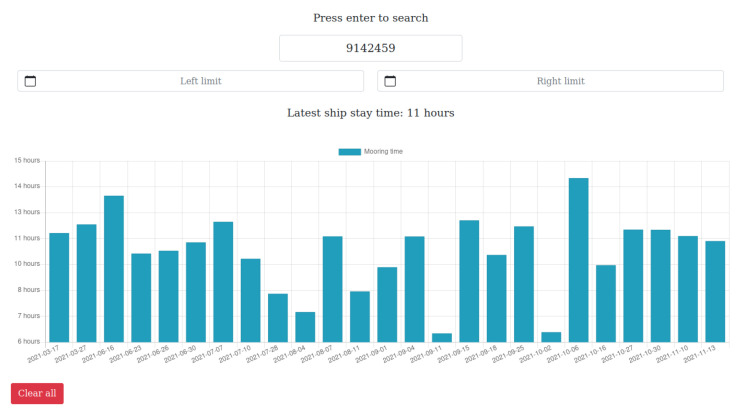
Ship Stay Time Web Interface.

**Figure 10:**
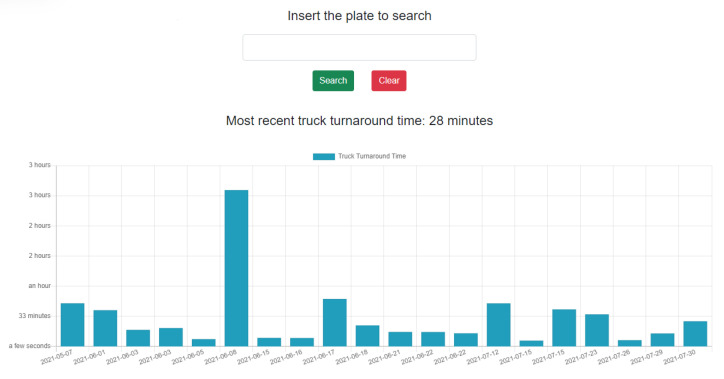
Truck Turnaround Time Web Interface.

One of the primary goals in the transition from the traditional port to the so-called Port of the Future is to provide easy access to the information submerged in a myriad of data already available to the Port Community System. This access can only be possible using a well-defined and standardized architecture. In Section 3, we thoroughly explored the proposed architecture, thus demonstrating its ability to be immediately adopted by any seaport, taking advantage of the prototype services described at the end of the section. The MONICA stack gives a jump start to the development, thanks to the following components: a DVL that unifies the way to access heterogeneous data coming from different sources; a standardized and common microservice development environment for repeatable, rapid prototyping; the authentication server which allows a centralized Single Sign-On (SSO) authentication schema. All these features allow the suggested architecture to be adopted as a reference architecture that can be integrated into any other port system, in order to obtain the same benefits described in the previous sections. Figure 7 and Figure 8 show the interface of the *Service A*: Enhanced awareness for vessel manoeuvres. Both real-time and static data are visible on the smart glasses. Figure 9 and Figure 10 show the results of *Services B* and *C*: the Ship Turnaround Time and Truck Turnaround Time. The interfaces allow users to visualize historical data in a bar chart. The stages of development to obtain the three prototypes are extremely similar, due to the adoption of the MONICA framework. As a result, little or no effort is spent making design choices, as the framework already does that for the developer. Furthermore, no matter how many developers work on creating applications on the stack, they will all be guided by the reference architecture, to achieve rapid prototyping and high maintainability of the developed artifacts.

## 5. Discussion

Currently, the port communities lack a common shared framework and architecture, designed with the inherent concept of multi-tenant infrastructure, enabling the development of innovative applications. Multiple service providers need to develop on the same stack, and often on the same cloud infrastructure. The test results of the performed experiments show that the architecture and framework presented totally cover the current needs of the port communities. These needs emerge when asking a group of independent providers to implement new functionalities. The solution that we propose, becomes the reference for anyone approaching, or that is already involved in, the Port of the Future: a unique, easy to adopt, scalable, and repeatable way of working that is fit for purpose. It is unique because the proposed solution encompasses the several scenarios present in every Port Community environment, from the physical IoT infrastructure to the end-user application layer. As the proposed MONICA architecture is well-tested, well-documented, and widely adopted in the Port of Livorno, it is guaranteed to be easily adopted by others. The core layers are intrinsically virtualized to be able to scale horizontally with ease. Finally, the three prototypes developed that we showed in Section 3 are the very example of the repeatability typical of such a kind of framework.

The ICT reference model adopted by the Port of Livorno fills most of the gaps presented in Section 1. Reorganizing to adopt a framework for the development and innovation of tools and applications fundamental for the Port of the Future, is the missing piece in a complex network of users interacting with the port ecosystem. Useless and harmful repetition and re-engineering go against many principles and practices of modern software development, hence, port communities should avoid the same antipatterns of the software industry. The benefits deriving from the adoption of the devised framework, are related to the port infrastructure as well as a heterogeneous group of roaming users (i.e., Vessels, Trucks, Trains, and their personnel) that daily interacts with it.

As a further step to expand the research, we aim at finding a different Port Community to test the framework on. The collected data and experience could significantly improve and stabilize the proposed work by enlarging the test set. In the scenario of a port adopting our framework, both parties could profit: on the one hand, the adopting parties can experience the same benefits that the Livorno port gained from using the MONICA stack. On the other hand, their feedback on the experience could help us improve and refine our research, so that many more institutions will be interested in adopting this innovative solution.

## 6. Conclusions

Port Community Systems are a fundamental means to connect private and public institutions to the port ecosystem. The potential offered by this platform is often limited by the lack of a standardized way to build on it. The proposed reference architecture, aims at providing a straightforward, easy-to-adopt solution to all the actors interested in the rapid development of service prototypes. In order to validate the proposed framework, we described three example services that have been implemented to demonstrate how the considered approach can support an agile development as well as integration of new and already existing services at the Port of Livorno. Rather than to forcibly adapt different scenarios, now the roaming users can use a unique reference to develop ICT services. As more and more port communities choose to adopt a standardized architecture, less time will be spent in the analysis and adaptation to several, port-specific technological stacks, and more will be dedicated to true innovation.

## Figures and Tables

**Table 1:** Classification of available framework based on addressed topic.

Framework	Addressed Topic
Minh et al. [12]	Investments & Situational Awareness
Ignaccolo et al. [13]	Sustainability
Bongdart et al. [14]	Sustainability
Acciaro et al. [15]	Sustainability
Stein et al. [16]	Sustainability
Bolognese et al. [17]	Sustainability & Situational Awareness
D’Amico et al. [18]	Sustainability & Situational Awareness
Schauer et al. [19]	Situational Awareness
Lacalle et al. [20]	Situational Awareness

## Data Availability

Raw data are sensitive information stored in a private cloud, property of the Port Authority of Livorno, and cannot be shared as open due to security reasons.

## References

[1] Moros-Daza A., Amaya-Mier R., Paternina-Arboleda C. (2020). Port Community Systems: A structured literature review. Transp. Res. Part A Policy Pract..

[2] Wilmsmeier G., Monios J., Lambert B. (2011). The Directional Development of Intermodal Freight Corridors in Relation to Inland Rerminals. J. Transp. Geogr..

[3] Pagano P., Antonelli S., Tardo A. (2021). C-Ports: A proposal for a comprehensive standardization and implementation plan of digital services offered by the “Port of the Future”. Comput. Ind..

[4] Notteboom T. (2004). Container Shipping And Ports: An Overview. Rev. Netw. Econ..

[5] Wilmsmeier G., Notteboom T. (2011). Determinants of liner shipping network configuration: A two-region comparison. GeoJournal.

[6] Notteboom T., Rodrigue J. (2005). Port Regionalization: Towards a New Phase in Port Development. Marit. Policy Manag..

[7] Monios J., Wilmsmeier G. (2013). The role of intermodal transport in port regionalisation. Transp. Policy.

[8] Molavi A., Lim G., Race B. (2019). A framework for building a smart port and smart port index. Int. J. Sustain. Transp..

[9] UNCTAD (1992). Port Marketing and the Challenge of the Third Generation Port. Report by Trade and Development Board. https://unctad.org/system/files/official-document/tdc4ac7_d14_en.pdf.

[10] UNCTAD The Fourth Generation Port. UNCTAD Ports Newsletter 1999. https://unctad.org/system/files/official-document/posdtetibm15.en.pdf.

[11] (2015). The Motorways of the Sea Digital Multi-Channel Platform. Smart Port. https://www.onthemosway.eu/wp-content/uploads/2015/07/1-Smart-Ports-v-2.0.pdf?00cab0.

[12] Minh Q., Sadiq R., Gucma L. (2021). Simulation-Based Performance Assessment Framework for Optimizing Port Investment. J. Waterw. Port Coast. Ocean Eng..

[13] Ignaccolo M., Inturri G., Giuffrida N., Torrisi V. (2020). A Sustainable Framework for the Analysis of Port Systems. Eur. Transp..

[14] (2019). Sustainable Urban Transport: Avoid-Shift-Improve (A-S-I). Transformative Urban Mobility Initiative. https://www.transformative-mobility.org/assets/publications/ASI_TUMI_SUTP_iNUA_No-9_April-2019.pdf.

[15] Acciaro M., Vanelslander T., Sys C., Ferrari C., Roumboutsos A., Lee J. (2014). Environmental sustainability in seaports: A framework for successful innovation. Marit. Policy Manag..

[16] Stein M., Acciaro M. (2020). Value Creation through Corporate Sustainability in the Port Sector: A Structured Literature Analysis. Sustainability.

[17] Bolognese M., Fidecaro F., Palazzuoli D., Licitra G. (2020). Port Noise and Complaints in the North Tyrrhenian Sea and Framework for Remediation. Environments.

[18] D’Amico G., Szopik-Depczyńska K., Dembińska I., Ioppolo G. (2021). Smart and sustainable logistics of Port cities: A framework for comprehending enabling factors, domains and goals. Sustain. Cities Soc..

[19] Schauer S., Rainer B., Museux N., Faure D., Hingant J., Carvajal Rodrigo F.J., Beyer S., Company Peris R., Zamarripa Lopez S. (2018). Conceptual Framework for Hybrid Situational Awareness in Critical Port Infrastructures. CRITIS 2018, Proceedings of the 13th International Conference, Kaunas, Lithuania, 24–26 September 2018.

[20] Lacalle I., Belsa A., Vaño R., Palau C.E. (2020). Framework and Methodology for Establishing Port-City Policies Based on Real-Time Composite Indicators and IoT: A Practical Use-Case. Sensors.

[21] Pagano P. (2016). Complex Infrastructures: The Benefit of ITS Services in Seaports. Intelligent Transportation Systems—From Good Practices to Standards.

[22] Cavalli L., Lizzi G., Guerrieri L., Querci A., De Bari F., Barbieri G., Ferrini S., Di Meglio R., Cardone R., Tardo A. (2021). Addressing Efficiency and Sustainability in the Port of the Future with 5G: The Experience of the Livorno Port. A Methodological Insight to Measure Innovation Technologies’ Benefits on Port Operations. Sustainability.

[23] Vermesan O. (2020). IoT Technologies for Connected and Automated Driving Applications. Internet of Things—The Call of the Edge-Everything Intelligent Everywhere.

[24] Teiid: Cloud-Native Data Virtualization. https://github.com/teiid/teiid.

[25] Teiid WildFly: Teiid Based on WildFly Application Server. https://teiid.github.io/teiid.io/teiid_wildfly/.

[26] Mobius OneM2M: OneM2M IoT Server Platform. https://github.com/IoTKETI/Mobius.

[27] OneM2M Sets Standards for the Internet of Things & M2M. https://www.onem2m.org/technical.

[28] TPCS-Tuscan Port Community System. http://www.tpcs.eu.

[29] Overview of the WSO2 API Gateway-API Manager Documentation 3.2.0. https://apim.docs.wso2.com/en/3.2.0/learn/api-gateway/overview-of-the-api-gateway/.

[30] RABBITMQ-Open Source Message Broker. https://www.rabbitmq.com/.

[31] OAuth 2.0-Industry Standard Protocol for Authorization. https://oauth.net/2/.

[32] OpenID Connect 1.0-[RFC6749] Protocol. https://openid.net/specs/openid-connect-core-1_0.html.

[33] JSON Web Token-Industry Standard RFC 7519. https://jwt.io/.

[34] Swagger. https://swagger.io/.

[35] VMWare ESXi. https://www.vmware.com/uk/products/esxi-and-esx.html.

[36] Kubernetes. https://kubernetes.io/docs/concepts/overview/what-is-kubernetes.

[37] Port Network Authority of the Northern Tyrrhenian Sea Archive of Port Authority Three-Year Master Plans. https://www.portialtotirreno.it/pianificazione-e-opere/piano-operativo-triennale/.

[38] The PortForward Project—Towards a Green and Sustainable Ecosystem for the EU Port of the Future. This Project Has Received Funding from the European Union’s Horizon 2020 Research and Innovation Programme under Grant Agreement No. 769267. https://www.portforward-project.eu/.

[39] Mobius-OCEAN DEVELOPERS. http://developers.iotocean.org/archives/module/mobius.

[40] OData-Standard Specification. https://www.odata.org/.

